# PH Domain-Arf G Protein Interactions Localize the Arf-GEF Steppke for Cleavage Furrow Regulation in *Drosophila*

**DOI:** 10.1371/journal.pone.0142562

**Published:** 2015-11-10

**Authors:** Donghoon M. Lee, Francisco F. Rodrigues, Cao Guo Yu, Michael Swan, Tony J. C. Harris

**Affiliations:** Department of Cell & Systems Biology, University of Toronto, Toronto, Ontario, Canada; National Cancer Institute, UNITED STATES

## Abstract

The recruitment of GDP/GTP exchange factors (GEFs) to specific subcellular sites dictates where they activate small G proteins for the regulation of various cellular processes. Cytohesins are a conserved family of plasma membrane GEFs for Arf small G proteins that regulate endocytosis. Analyses of mammalian cytohesins have identified a number of recruitment mechanisms for these multi-domain proteins, but the conservation and developmental roles for these mechanisms are unclear. Here, we report how the pleckstrin homology (PH) domain of the *Drosophila* cytohesin Steppke affects its localization and activity at cleavage furrows of the early embryo. We found that the PH domain is necessary for Steppke furrow localization, and for it to regulate furrow structure. However, the PH domain was not sufficient for the localization. Next, we examined the role of conserved PH domain amino acid residues that are required for mammalian cytohesins to bind PIP3 or GTP-bound Arf G proteins. We confirmed that the Steppke PH domain preferentially binds PIP3 *in vitro* through a conserved mechanism. However, disruption of residues for PIP3 binding had no apparent effect on GFP-Steppke localization and effects. Rather, residues for binding to GTP-bound Arf G proteins made major contributions to this Steppke localization and activity. By analyzing GFP-tagged Arf and Arf-like small G proteins, we found that Arf1-GFP, Arf6-GFP and Arl4-GFP, but not Arf4-GFP, localized to furrows. However, analyses of embryos depleted of Arf1, Arf6 or Arl4 revealed either earlier defects than occur in embryos depleted of Steppke, or no detectable furrow defects, possibly because of redundancies, and thus it was difficult to assess how individual Arf small G proteins affect Steppke. Nonetheless, our data show that the Steppke PH domain and its conserved residues for binding to GTP-bound Arf G proteins have substantial effects on Steppke localization and activity in early *Drosophila* embryos.

## Introduction

The recruitment and activation of GDP/GTP exchange factors (GEFs) at specific sites is critical for the activation of small G proteins. These small G proteins control a wide range of cellular processes, including growth, cytoskeletal dynamics and membrane trafficking [1, 2]. Plasma membrane (PM) Arf small G proteins are major, direct inducers of endocytosis and related signaling [3–7]. Both Arf6 and Arf1 act at the PM, whereas Arf1, Arf4 and others induce membrane budding from internal membrane compartments, such as the Golgi. Like other G proteins, they are activated by GEFs and inactivated by GTPase activating proteins (GAPs). In humans, 11 Arf-GEFs are predicted to act at the PM and form three main groups; cytohesins, EFA6s, and BRAGs [3]. In *Drosophila*, the situation is simpler, with just one GEF per group; Steppke (Step; a cytohesin), EFA6, and Loner/Schizo (a BRAG) [3]. Notably, cytohesins, EFA6s and BRAGs have been shown to act on both Arf6 and Arf1 [3, 4, 8, 9], and BRAG2 also promotes endocytosis via Arf5 [10].

Our research focuses on the cytohesin Step and how it regulates cleavage of the early *Drosophila* embryo. *Drosophila* embryogenesis begins as a syncytium in which PM cleavage furrows transiently separate dividing somatic nuclei and then fully cellularize ~6000 nuclei to form the cellular blastoderm [11–13]. Step localizes to these cleavage furrows and uses its Arf-GEF activity to antagonize the Rho1-actomyosin pathway during two forms of cell division [14, 15]. Step acts first to control primordial germ cell division from the syncytial soma, and without Step, the division occurs at ectopic sites [15]. Then, Step continues to act during the syncytial nuclear divisions of the soma to prevent closure of cleavage furrows at their basal tips. Without Step, basal membranes form abnormally creating cells prematurely or displacing nuclei from the embryo periphery [14]. Suggesting a conserved role for cytohesins in the regulation of cell division, the *C*. *elegans* cytohesin localizes to neuroblast cytokinetic furrows and regulates their asymmetric division [16]. In *Drosophila*, a conserved cytohesin adaptor, Stepping stone (Sstn; a distant homolog of mammalian FRMD4A), aids Step in its antagonism of actomyosin networks. Sstn and Step interact directly through their coiled-coil (CC) domains, as mammalian FRMDA4 and cytohesins do [17]; the proteins co-localize at cleavage furrows; and depletion of Sstn partially phenocopies the effects of *step* loss [18]. However, Sstn is not solely responsible for Step recruitment and activation at the cleavage furrows [18].

Mammalian cell culture and biochemical studies have identified many mechanisms for recruiting and activating cytohesins at the PM through their N-terminal CC domain and their C-terminal pleckstrin homology (PH) domain [3, 4]. These mechanisms are linked to the direct output of cytohesins—Arf activation mediated by the central Sec7 GEF domain. Cytohesin CC domains can bind various adaptor proteins to integrate cytohesins with different PM complexes [17, 19–24]. Additionally, the CC domain can reversibly block the PH domain to control membrane association [25], and can dimerize [26, 27]. When free, the PH domain of cytohesins can bind PM PIP2 and PIP3, or PIP3 preferentially, depending on a tri-glycine or di-glycine repeat in different isoforms [28, 29]. Additionally, a separate face of the PH domain can bind to GTP-bound Arf6 [26, 30] and GTP-bound Arf-like 4 (Arl4) [31]. These interactions, as well as phosphorylation events, have been shown to relieve inhibitory binding of the Sec7 domain by the PH domain [26], creating a potential positive feedback loop with cytohesins producing Arf-GTP and Arf-GTP activating cytohesins [32]. Overall, localized cytohesin activity seems to be controlled by multiple direct mechanisms, but it is unclear whether these activities act together in different contexts or how they function during development.

Since Sstn was identified as an interaction partner for the Step CC domain [18], we turned to its PH domain to understand how Step is recruited and activated at *Drosophila* cleavage furrows. The PH domain of Step has been used as a probe for PIP3 (named tGPH) [33]. It was shown to respond to PIP3 production *in vivo* [33] and to bind PIP3 liposomes *in vitro* [34]. However, reduction of PIP3 seems to have no effect on early cleavage furrows of the *Drosophila* embryo, despite a specific and strong effect during mid-late cellularization [35]. Thus, it was unclear whether and how the PH domain would affect Step localization and activity at early *Drosophila* cleavage furrows.

Here, we show that the Step PH domain makes an important contribution to both the localization and activity of Step at *Drosophila* cleavage furrows, apparently through PH domain interactions with Arf small G proteins.

## Results

To identify mechanisms of Step localization and activation, we conducted a structure-function analysis focused on the PH domain of Step. We created constructs in which the PH domain or other domains were deleted, or in which conserved amino acid residues shown to mediate specific interactions in mammalian studies were changed to non-functional residues based on these same studies. The constructs were UAS-controlled, inserted at the same chromosomal site (confirmed by PCR), and GFP-tagged. To analyze their localization and activity in early embryos, we expressed them maternally and imaged progeny as syncytial embryos when maternal supplies direct development.

### The Step PH domain is necessary but not sufficient for Step localization

**Fig 1:**
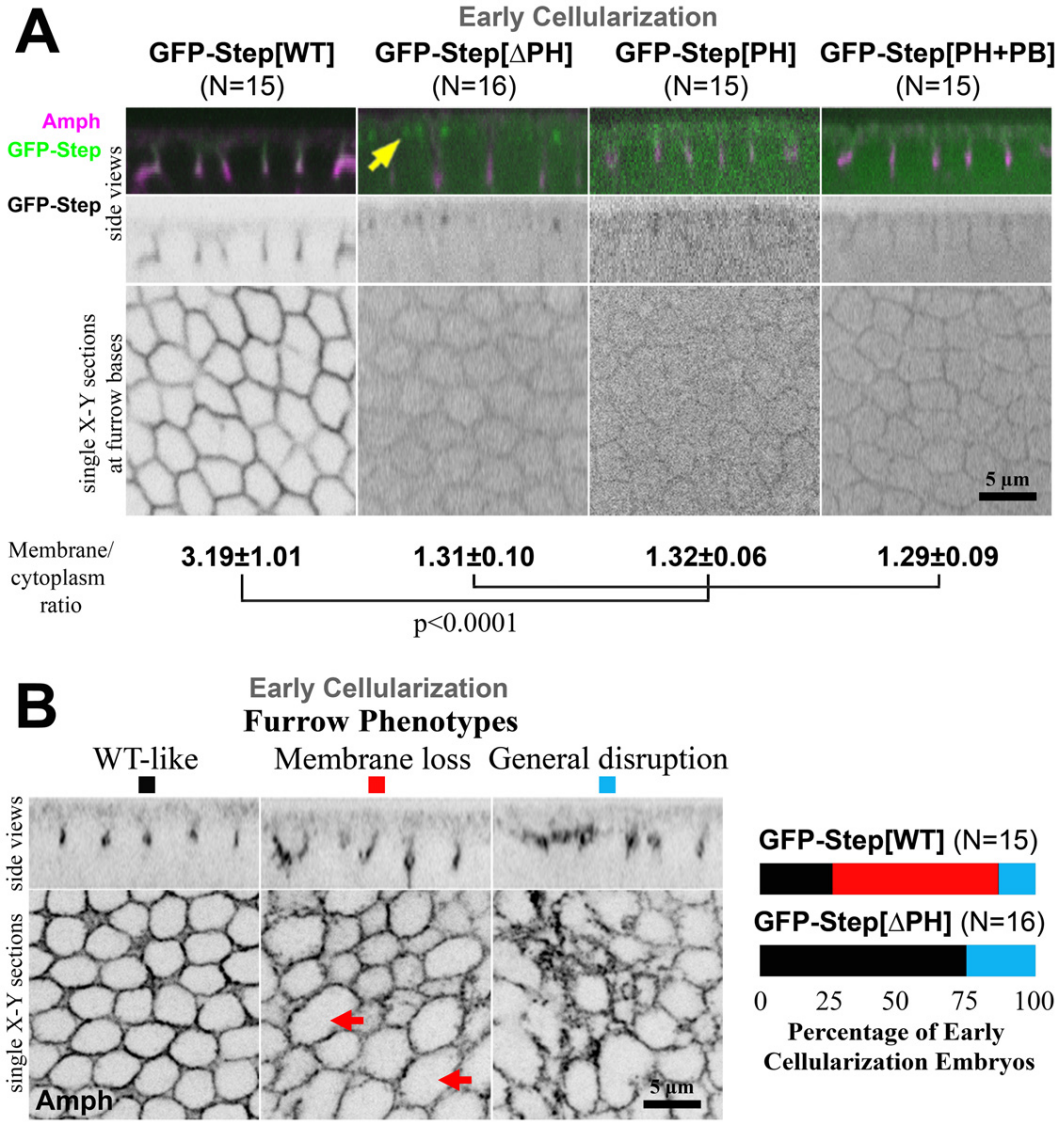
The PH domain of Step is necessary but not sufficient for its membrane localization and activity. (A) Localization of GFP-Step constructs at early cellularization. N values indicate the number of embryos analyzed quantitatively. Amphiphysin (Amph) staining indicates the furrows. GFP-Step[WT] had a higher furrow membrane:cytoplasm ratio than the other constructs, as shown in the micrographs and quantifications of the ratios (means ± SD). GFP-Step[∆PH] also localized to structures that appeared to be centrosomes (yellow arrows). (B) GFP-Step[WT] over-expression produced a range of effects on furrows at early cellularization, from no effect (black) to sporadic membrane loss (red, arrows) to a general disruption of furrows including membrane loss and other disorganization (blue). Quantification of furrow defects (right) showed that the GFP-Step[∆PH] construct had a weaker effect on furrows than GFP-Step[WT] (N represents embryo numbers).

Since the PH domain of cytohesins aids their membrane recruitment, we investigated how the PH domain of Step affects its localization to cleavage furrows in the cellularizing *Drosophila* embryo. At this stage, localization data for Step is limited to the imaging of a full length GFP-Step construct since an antibody shown to specifically detect Step by immunofluorescence of imaginal discs [24] cannot detect Step above background in the syncytial embryo suggesting relatively low protein expression [15]. Importantly, an RNAi-resistant form of the GFP-Step construct has been shown to rescue *step* RNAi embryos [14, 15], indicating that GFP-Step can replace the activity of endogenous Step, although its overexpression with the Gal-4-UAS system remains a caveat. GFP-Step localizes to the PM furrows with enrichment at their basal tips (Fig 1A) [14], the PM sites that display the greatest mis-regulation with experimental Step removal [14]. To determine the role of the PH domain in this localization, we imaged GFP-Step[∆PH] and found that it had a much greater cytoplasmic pool resulting in a lower PM:cytoplasm localization ratio (Fig 1A), and additionally localized to two sub-apical foci per cell typical of a centrosome association (Fig 1A, arrow). To test whether the PH domain is sufficient for the full length GFP-Step localization pattern, we imaged GFP-Step[PH], GFP linked to the PH domain alone, and found that it too had a much lower PM:cytoplasm localization ratio than full length Step (Fig 1A). It was possible that the C-terminal poly-basic region was needed for PH domain localization, but imaging of a well-characterized construct containing both regions, GFP-Step[PH+PB] (the tGPH probe [33]), revealed a distribution similar to the PH domain alone. Thus, the PH domain of Step is needed for its full PM association, but the domain alone is not sufficient for this localization.

### The Step PH domain is necessary for full Step activity

Previously, we found that Step over-expression leads to sporadic furrow loss (the opposite of the abnormal furrow expansion that occurs with experimental Step removal), and that this effect was dependent on Step’s GEF activity [14]. Thus, we used this over-expression effect as an assay for Step construct activity. First, we compared full length GFP-Step and GFP-Step[∆PH]. The majority of embryos overexpressing full length GFP-Step displayed either sporadic loss of furrow membranes (Fig 1B, arrows) or a general disruption to furrows across the embryo (involving both furrow loss and furrow disorganization; Fig 1B). In contrast, the majority of embryos expressing GFP-Step[∆PH] displayed normal furrows (Fig 1B, right graph). Thus, the PH domain of Step is also needed for it to affect the PM in this assay.

### The sequence and lipid binding properties of the Step PH domain are conserved

**Fig 2:**
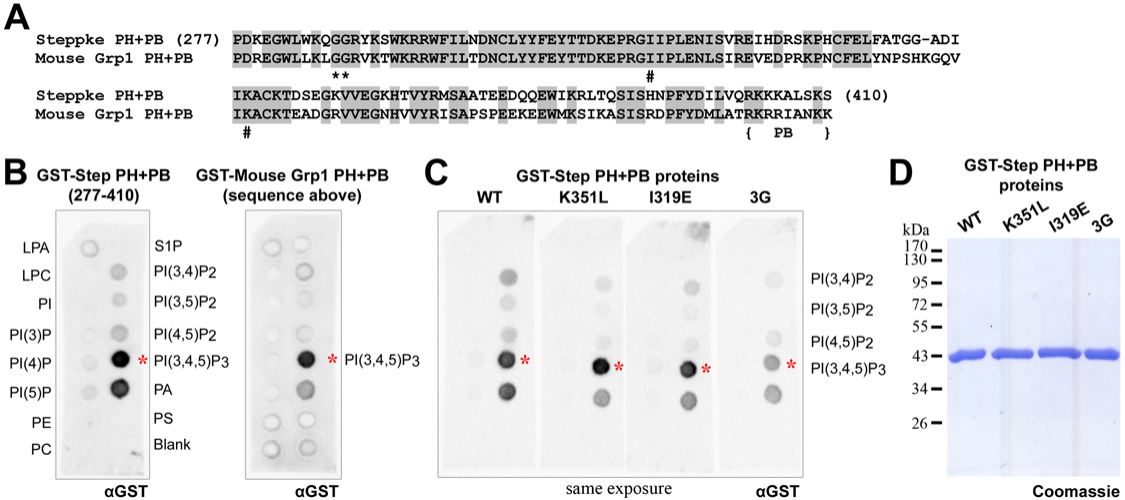
Conserved sequence and lipid binding properties of the PH domain of Step. (A) The sequence of the PH domain and PB region of Step compared to that of mouse Grp1. The di-glycine sequence implicated in PIP3 binding is marked with asterisks. The Ile and Lys residues implicated in GTP-bound Arf small G protein binding are marked with pound symbols. (B) The amino acid sequences in (A) were expressed as GST fusions and exposed to lipid arrays at 0.08 μg/ml. Both showed similar preferential binding to PIP3 (red asterisks). (C) The PIP3 binding of the GST Step PH+PB protein was unaffected by amino acid residue changes expected to disrupt GTP-bound Arf small G protein binding and was lowered by conversion of the di-glycine sequence to a tri-glycine sequence (red asterisks). The proteins were exposed to lipid arrays at 0.08 μg/ml and the arrays were processed and imaged side-by-side (shown as a single photograph of all four arrays). Similar amounts and stabilities of the proteins were confirmed by separating 3 μg of each protein by SDS-PAGE and performing a Coomassie stain (D).

Since specific residues of the PH domains of mammalian cytohesins have been implicated in binding either phosphoinositide species or GTP-bound Arf small G proteins, we compared the sequence of the Step PH domain to those of its mammalian counterparts. As shown for a comparison with the mouse Grp1 PH domain, the Step PH domain has high sequence identity with these domains (Fig 2A). In particular, Step contains the di-glycine motif that conveys specificity for PIP3 (Fig 2A, asterisks, [28, 29]), as well as conserved residues implicated in binding to GTP-bound Arf small G proteins, I319 and K351 (Fig 2A, pound symbols, [30, 31]).

The PH domain of mammalian Grp1 binds PIP3 specifically and is a commonly used probe for PIP3 *in vivo* [36]. The PH domain of Step has been shown to respond to production of PIP3 *in vivo* [33], and to bind PIP3 vesicles *in vitro* [34], but its relative affinity for PIP3 and PIP2 species has not been reported. Thus we compared the binding of equimolar GST-Step[PH+PB] and GST-mouse Grp1[PH+PB] to an array of lipid species immobilized on nitrocellulose. Both proteins displayed similar strong and specific binding to PIP3 in repeated experiments (Fig 2B, red asterisks). To test if similar residues are involved in this interaction we converted the Step di-glycine motif to the tri-glycine motif found in natural mammalian cytohesin isoforms [28, 29]. This alteration weakened the binding of GST-Step[PH+PB] to PIP3 in repeated side-by-side assays (Fig 2C, red asterisks). In contrast, mutations shown to weaken binding to GTP-bound Arf G proteins (I319E and K351L) had no effect on the binding of GST-Step[PH+PB] to PIP3 or other lipids (Fig 2C, red asterisks), as observed in binding studies of the mammalian proteins [37]. Together, these sequence comparisons, sequence perturbations and lipid binding assays suggest that the PH domain of Step is very similar to its mammalian counterparts.

### Disrupting residues for Arf-GTP binding weakens Step membrane association, but disrupting PIP3 binding does not

**Fig 3:**
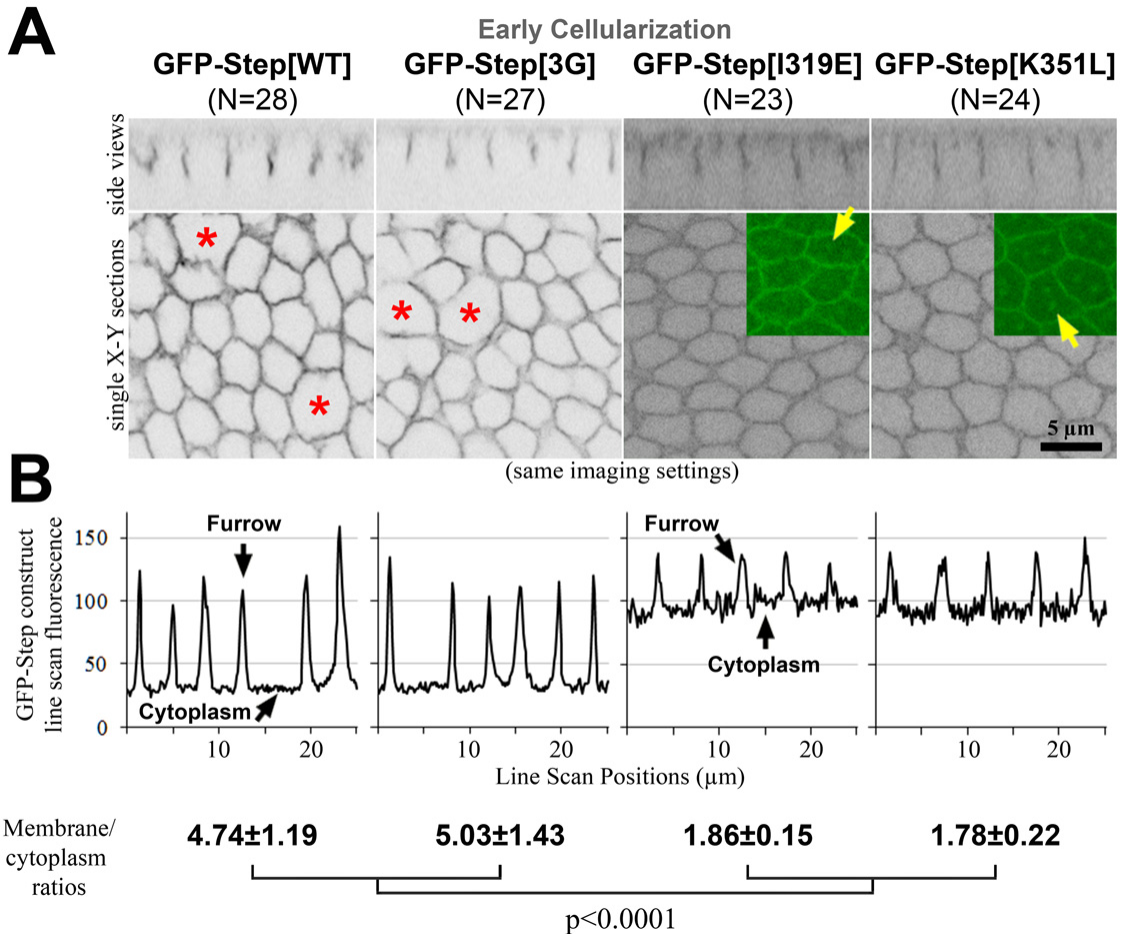
Amino acid residue changes affecting the localization of Step. GFP-Step[WT] and GFP-Step[3G] had indistinguishable furrow localization (assessed as in Fig 1A, and additionally with line scans). Over-expression of either protein also induced sporadic furrow loss (red asterisks). GFP-Step[I319E] and GFP-Step[K319L] showed a lower furrow membrane:cytoplasm ratio (quantified as in Fig 1A), and also localized to structures that appeared to be centrosomes (yellow arrows).

Since the PH domain of Step is well conserved, we hypothesized that one or more of the interactions identified for the mammalian domain would affect Step furrow localization and activity. Thus, we generated UAS constructs to express the following proteins *in vivo*: GFP-Step[3G] to reduce PIP3 binding; and GFP-Step[I319E] and GFP-Step[K351L] to reduce binding to GTP-bound Arf G proteins. Imaging these three proteins and full length GFP-Step with the same settings revealed an indistinguishable distribution of full length GFP-Step and GFP-Step[3G] at early cellularization, whereas both GFP-Step[I319E] and GFP-Step[K351L] displayed a much greater cytosolic pool (Fig 3A, quantified in 3B), as well as localization to what appeared to be centrosomes (Fig 3A arrows). To confirm the minimal effect of the mutation affecting PIP3 binding, we generated a Step construct with mutation of another conserved residue for PIP3 binding (GFP-Step[R286C] [37]), and its localization was also indistinguishable from full length GFP-Step (data not shown). Thus, the mutation of residues implicated in binding to GTP-bound Arf G proteins had a similar effect on Step localization as deleting the full PH domain (Fig 1), whereas weakening PIP3 binding had no detectable effect. These results do not exclude a role for PIP3 binding since the altered protein has residual PIP3 binding activity (Fig 2C) and could also localize through homo-oligomerization with endogenous Step in the system [18]. However, the results do implicate a role for binding to GTP-bound Arf G proteins.

**Fig 4:**
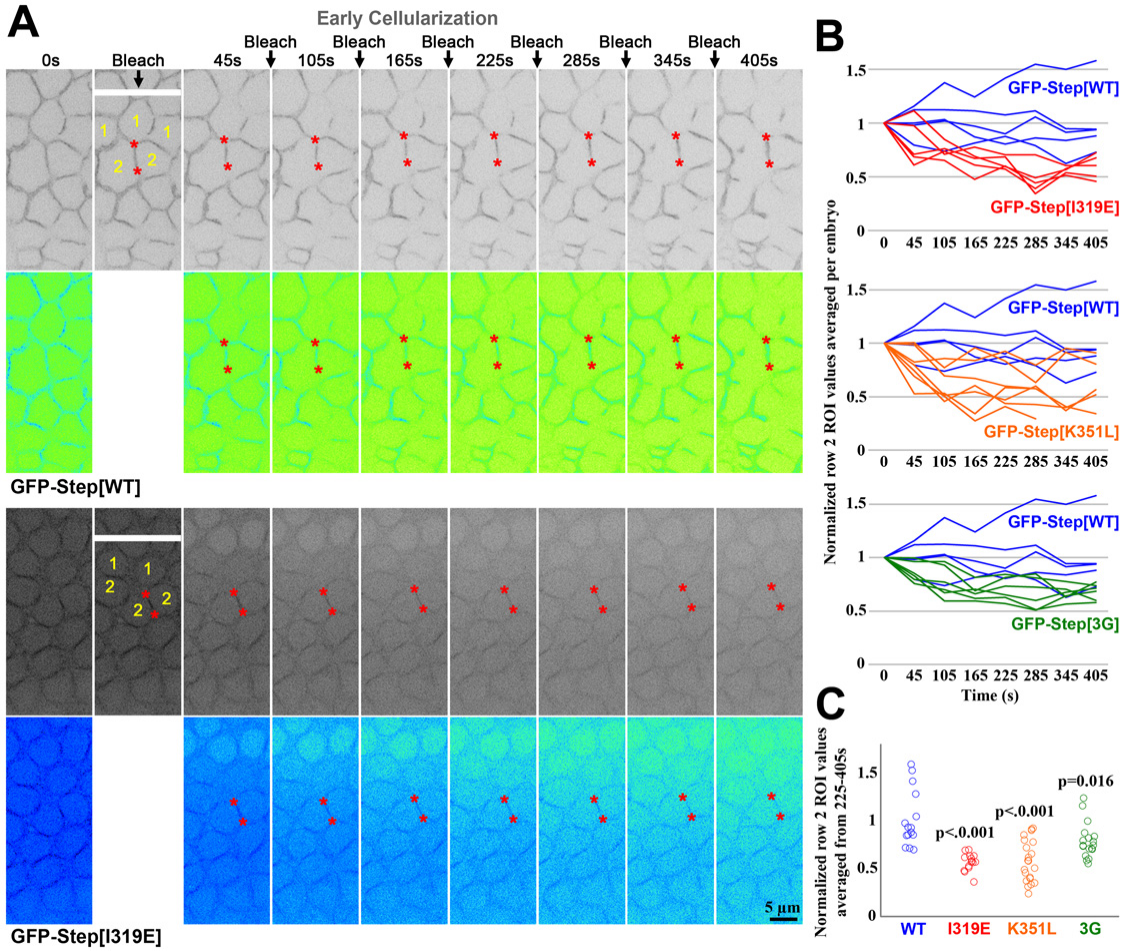
Amino acid residue changes affecting the maintenance of Step at furrow membranes. (A) Time points from FLIP analyses of GFP-Step[WT] and GFP-Step[I319E] at early cellularization. 0s shows the embryo before the first photobleaching. In the second column, the white bar shows where the embryo was photobleached, as well as cell compartments in Row 1 and Row 2 away from the cell row being bleached. The same positions were bleached at 60s intervals (arrows), and the embryos are shown 45s after each bleaching. The red asterisks indicate example Row 2 furrows that maintain their signal for GFP-Step[WT] or become depleted of signal for GFP-Step[I319E]. The images are also shown with an inverted Fire look up table (Image J) to show that a gradient of both cytosolic and membrane signal depletion arises with the repeated GFP-Step[I319E] photobleaching, but not for GFP-Step[WT]. (B) Quantification of the responses at Row 2 furrows as averages of three sites per embryo normalized to the signals at 0s (each line is data from one embryo). (C) Quantification of the same responses at Row 2 furrows as in B, but as averages of each single site over time points 225-405s normalized to the signals at 0s (each circle is data from one furrow). For B and C, note that Step[I319E] displayed the greatest increase in mobility versus Step[WT].

We hypothesized that the greater cytosolic pool of GFP-Step[I319E] and GFP-Step[K351L] versus GFP-Step[WT] and GFP-Step[3G] might reflect a decrease of protein maintenance at furrow membranes. To investigate this possibility more directly, we conducted fluorescence loss induced by photobleaching (FLIP) experiments. Since the cellular compartments of the early cellularization embryo have a shared cytoplasm, we bleached a narrow rectangle covering the middle of a central row of cells at one minute intervals, and then monitored the behavior of GFP-Step constructs in the first, second and further rows of cells away from those being repeatedly bleached. For GFP-Step[WT], the effects of the repeated bleaching were relatively local over a 6 minute period. Specifically, the cell row being bleached showed a reduction of signal, but no gradient of depleted signal was observed over the neighboring and more distant rows, and quantification of the second neighboring row revealed no decrease in signal (after correction for the general photobleaching from the imaging laser) (Fig 4A–4C). The behavior of the GFP-Step[3G] construct was most similar to the full length, perhaps with somewhat greater mobility (Fig 4B and 4C). In contrast, both GFP-Step[I319E] and GFP-Step[K351L] displayed greater mobility through the syncytial embryo, with GFP-Step[I319E] being most mobile. Specifically, the cell row being bleached showed a reduction of signal, a gradient of depleted signal was observed both at membranes and in the cytoplasm over the neighboring and more distant rows, and quantification of the second neighboring row revealed significant decreases in signal in response to the repeated photobleaching (Fig 4A–4C, red asterisks). Thus, the maintenance of GFP-Step at membrane furrows has a specific dependence on residues implicated in binding to GTP-bound Arf G proteins.

### Disrupting residues for Arf-GTP binding weakens Step membrane activity, but disrupting PIP3 binding does not

**Fig 5:**
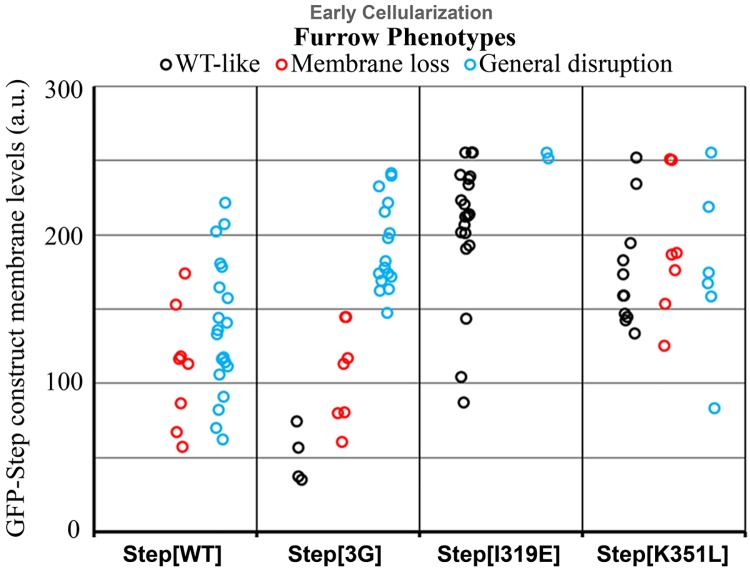
Amino acid residue changes affecting the activity of Step. GFP-Step[WT] and GFP-Step[3G] had indistinguishable disruptive effects on furrows (assessed as in Fig 1B). GFP-Step[I319E] and GFP-Step[K351L] each showed a lower degrees of furrow disruption, despite similar furrow levels as GFP-Step[WT]. The difference was greater for GFP-Step[I319E].

To determine how the specific residues within the Step PH domain affect the activity of Step, we compared their membrane levels and effects on membrane furrow organization. Both full length GFP-Step and GFP-Step[3G] lead to similar degrees of furrow loss and general membrane disruption with similar levels of protein at the furrows (Fig 3A, red asterisks, Fig 5, quantification), as did GFP-Step[R286C] (data not shown). In contrast, both GFP-Step[I319E] and GFP-Step[K351L] displayed minimal activity despite equal or greater protein levels at the furrows (Fig 5). Thus, the activity of GFP-Step at membrane furrows also has a specific dependence on residues implicated in binding to GTP-bound Arf G proteins.

### Arf6, Arf1 and Arl4 are candidate regulators of Step

**Fig 6:**
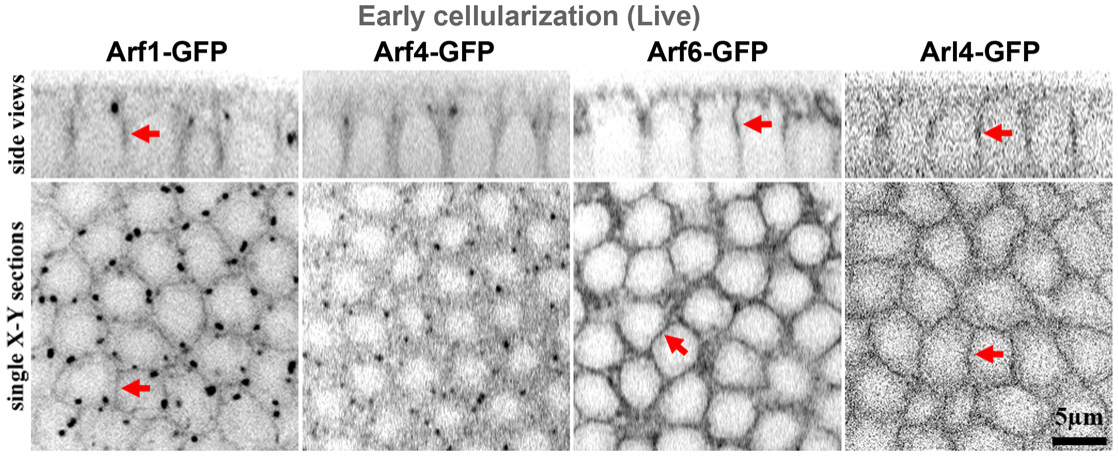
Localization of GFP-tagged Arf and Arf-like small G proteins at early cellularization. Live imaging revealed Arf1-GFP, Arf6-GFP and Arl4-GFP localization to furrows (arrows). Arf1-GFP also localized to strong cytosolic puncta resembling Golgi, and Arf4-GFP did as well.

Since our data implicated binding to GTP-bound Arf G proteins for the membrane maintenance and activity of Step, we investigated which Arf or Arf-like G proteins might be involved. First, we investigated the localization of the three *Drosophila* Arf G proteins (Arf1, Arf4, and Arf6) and the Arf-like protein implicated in cytohesin recruitment (Arl4) by generating constructs for expressing GFP fusion proteins from UAS transgenes *in vivo*. During early cellularization, Arf1-GFP localized moderately to the PM furrows as well as strongly to internal puncta (Fig 6) that were positive for Golgi markers (data not shown); Arf4-GFP was only detected at strong puncta resembling those positive for Arf1-GFP (Fig 6); Arf6-GFP localized strongly to the PM furrows (Fig 6); and Arl4-GFP localized weakly to the PM furrows (Fig 6). Thus, Arf1, Arf6 and Arl4 localize to furrows where they could possibly maintain Step.

**Fig 7:**
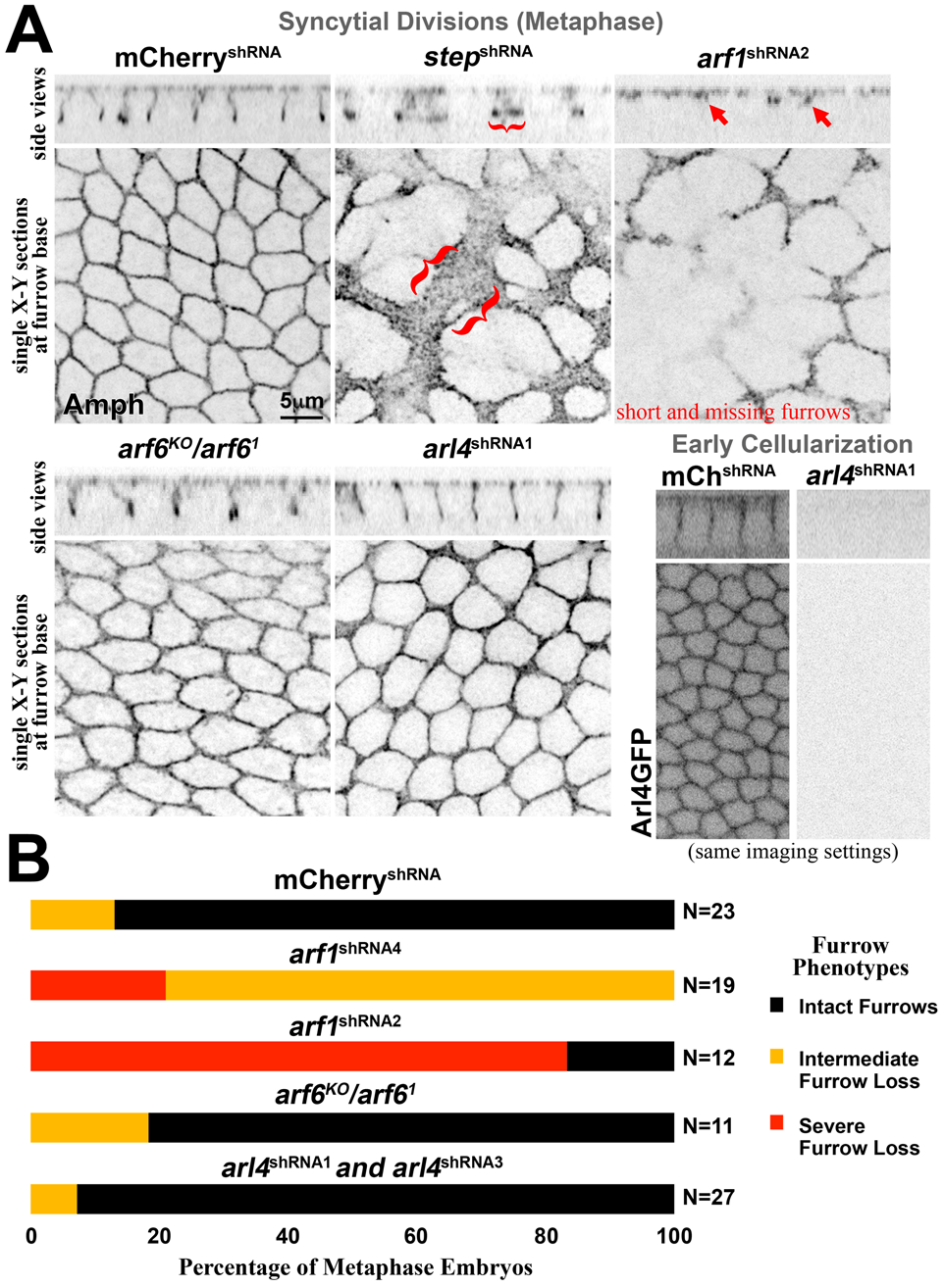
The effects of Arf or Arf-like small G protein removal are distinct from those of Step removal. Embryos with peripherally dividing nuclei at metaphase were identified by phospho-histone H3 staining (not shown). (A) Amph staining shows the intact furrows of a control (mCherry) RNAi embryo and the expansion of the furrow base (brackets) that occurs with *step* RNAi. *arf1* RNAi resulted in furrow loss (arrows; note that the X-Y section showing the base of furrows is closer to the embryo surface in this case). *arf6* null mutants showed no apparent defects, and *arl4* RNAi embryos displayed no apparent defects despite an ability of the RNAi to effectively deplete Arl4-GFP (shown for early cellularization). (B) A quantification of the furrow phenotypes with Arf and Arf-like small G protein depletion. Two different *arf1* shRNA lines produced similar defects with different strengths (data shown separately). Two different *arl4* shRNA that each effectively depleted Arl4-GFP had indistinguishable effects on furrows (data combined). N represents embryo numbers.

Removal of Step, either with *step* RNAi or mutants, leads to premature cell formation through the abnormal expansion of basal membranes [14] (Fig 7A). To test whether Arf1, Arf6 or Arl4 aid Step to prevent this abnormal membrane change, we targeted them individually through maternal loss-of-function approaches. Maternal depletion of Arf1 by RNAi resulted in major fertility defects, and the embryos that were produced displayed a loss of cleavage furrows (Fig 7A and 7B) that might be associated with the role of Arf1 in biosynthetic trafficking from the Golgi [3, 4, 11]. *arf6* null mutants are adult viable and fertile as females [38, 39] and we observed no apparent defects in cleavage furrows derived from these females (Fig 7A and 7B), although patches of nuclear loss (nuclear fall-out) were observed in some of embryos (data not shown). To test the role of Arl4, we constructed shRNA constructs targeting Arl4 and found that two effectively eliminated Arl4-GFP by microscopy (Fig 7A), but they had no apparent effect on early cellularization furrows (Fig 7A and 7B). Finally, the effects of *arl4* RNAi in *arf6* null mutants were indistinguishable from the effects of control mCherry RNAi in the *arf6* null mutants (data not shown), suggesting there is not substantial redundancy between these proteins. Three non-mutually exclusive possibilities could reconcile these observations with our structure-function analyses of the Step PH domain: (i) a subset of Arf1 might activate Step, but this effect is masked by the earlier developmental disruptions of *arf1* RNAi, (ii) there is redundancy between Arf1 and the other proteins, or (iii) there is redundancy with other Step activation mechanisms.

## Discussion

Our data identify a role for the PH domain of Step for its localization and activity at cleavage furrows of the early *Drosophila* embryo. More specifically, conserved residues for PH domain-Arf-GTP binding make a substantial contribution to Step membrane association and control.

A role for PH domain-Arf-GTP binding in the localization and activation of Step provides a mechanistic explanation for two recent findings in the early *Drosophila* embryo. First, Sstn was discovered as a Step adaptor protein, as it binds Step directly through coiled-coil domain hetero-dimerization and can recruit Step to the PM, but was not the sole molecule needed for Step localization and activity, as cleavage furrow mis-regulation was weaker with *sstn* RNAi versus *step* RNAi, and Step over-expression lead to furrow localization and control even with *sstn* RNAi [18]. Our current results suggest that the PH domain of Step might be able to convey Step activity in the absence of Sstn. Second, PIP3, a molecule known to bind to the PH domain of Step and recruit it to membranes (Fig 2 [33, 34]), was shown to be unnecessary for early cleavage furrow regulation in *Drosophila* [35]. Together with our data, this suggests that PH domain-Arf-GTP binding may be more significant than PH domain-PIP3 binding for Step recruitment and activity at early *Drosophila* cleavage furrows.

The source of Arf-GTP for Step recruitment then becomes the question. First, which Arf or Arf-like small G proteins are involved? Based on GFP-fusion protein localization, it appears that Arf1, Arf6 or Arl4 are in position to impact Step. However, other roles of these Arf/Arf-like G proteins and possible redundancies between them make it difficult to test for effects on Step localization and activity through loss-of-function experiments. Second, how would the Arf/Arf-like G proteins be converted to their GTP-bound form to initiate Step activation? It is possible that *Drosophila* EFA6 or the *Drosophila* BRAG (Loner/Schizo) catalyze GDP-GTP exchange for Arf1 or Arf6 [3, 4, 8, 9], as both GEFs are expressed during early embryogenesis (FlyBase). However, neither GEF has been characterized rigorously at this stage, and EFA6 null mutant females were shown to be viable and fertile [38]. With some Step activation, Step could additionally catalyze GDP-GTP exchange on Arf1 or Arf6 as part of a proposed positive feedback loop [32]. For Arl4, GDP-GTP exchange may occur intrinsically. Although most small G proteins have negligible rates of nucleotide exchange and GTP hydrolysis on their own, a group of Arf-like proteins (Arl4, Arl6, and Arl7) has been shown to undergo intrinsic GDP-GTP exchange [40], and there have been no reports of GEFs for Arf-like G proteins [4]. *Drosophil*a Arl4 may be controlled in a similar manner, as it is 63% identical to the human Arl4 protein. These mechanisms, GEF-produced Arf1-GTP and Arf6-GTP and intrinsically-produced Arl4-GTP, may provide sources of Arf-GTP for Step PM recruitment and activation.

Our study provides new mechanistic insight into the Step pathway for Rho1-actomyosin regulation during *Drosophila* embryonic cleavage. It appears that a conserved PH domain-Arf-GTP interaction contributes to Step PM association and activity. As cautioned from the studies of mammalian cytohesins [26, 30, 31], these interactions should also be taken into consideration when using the PH domain of Step as an *in vivo* probe for PIP3.

## Materials and Methods

### Molecular biology and transgenics

#### GFP-Step constructs

Previously generated UASp-GFP-Step[WT] [14] was used as a template with mutagenesis of the *step* sequence in the gateway entry vector (Invitrogen) prior to recombination into the gateway destination vector pPGW (Invitrogen) for an upstream UASp sequence containing and N-terminal EGFP tagging. For UASp-GFP-Step[∆PH], the template was mutated by PCR with an established protocol [14] to create HindIII restriction sites at both ends of the PH domain, using 5'-CTTCAATCCCGACAAGCTTGGCTGGCTGTGG-3' (forward1), 5'-CCACAGCCAGCCAAGCTTGTCGGGATTGAAG-'3 (reverse1), 5’-GGCACTCAGCAAGCTTTAATAGCGGCCGC-3’ (forward2) and 5’-GCGGCCGCTATTAAAGCTTGCTGAGTGCC-3’ (reverse2). The PCR product was cut with HindIII and re-ligated to generate the final construct without the PH domain. For UASp-GFP-Step[3G], UASp-GFP-Step[R286C], UASp-GFP-Step[I319E] and UASp-GFP-Step[K351L], the template was mutated by PCR with the following respective primers: 5’-CTGGCTGTGGAAGCAAGGAGGCGGCAGATACAAATCG-3’ (forward) and 5’-CGATTTGTATCTGCCGCCTCCTTGCTTCCACAGCCAG-3’ (reverse), using 5’- caaatcgtggaaacgatgctggttcattttg-3’ (forward) and 5’- caaaatgaaccagcatcgtttccacgatttg-3’ (reverse), 5’-GAACCACGCGGAGAAATACCGCTGGAG-3’ (forward) and 5’-CTCCAGCGGTATTTCTCCGCGTGGTTC-3’ (reverse), and 5’-GGTGCTGATATAATCCTGGCATGCAAGACTG-3’ (forward) and 5’-CAGTCTTGCATGCCAGGATTATATCAGCACC-3’ (reverse). For UASp-GFP-Step[PH], the nucleotide sequences of the Step PH domain (cag[CTGACA…cDNA of the Step PH domain…GAATAA]TAGCGGCCGC) were constructed by gene synthesis technology (GenScript Inc), cut by PvuII and NotI, and cloned into gateway entry vector (Invitrogen) at EheI and NotI sites for subsequent recombination into pPGW. The destination vectors for GFP-Step[WT], UASp-GFP-Step[3G], UASp-GFP-Step[R286C], UASp-GFP-Step[I319E] and UASp-GFP-Step[K351L] were inserted into the genome at the attP40 recombination site on the second chromosome, and the vector for GFP-Step[PH] was targeted to the attP2 recombination site on the third chromosome (BestGene Inc).

#### GFP-Arf and -Arl constructs

For UASp-Arf4-GFP, the *arf4* coding sequence was PCR amplified from cDNA (RE53354; the Canadian Drosophila Microarray Centre (CDMC)), using 5’-AGATATGTCGACATTAATCGAATGACTAG-3’ (forward) and 5’-AGTGTTCTCGAGCCTTTTTTAGCCAATTCAGC-3’ (reverse) primers. For UASp-Arl4::GFP, the *arl4* coding sequence was PCR amplified from cDNA (AT26185; CDMC), using 5’-AGATATGTCGACAAAATCGACAATATTT-3’ (forward) and 5’-AGTGTTCTCGAGAGATTTCTTTTATTTG-3’ (reverse) primers. The PCR products were cloned into gateway entry vector (Invitrogen) at SalI and XhoI sites, and recombined into gateway destination vector pPWG (Invitrogen) containing a C-terminal EGFP tagging and an upstream UASp sequence. The destination vectors for Arf4-GFP was inserted into the genome using P-element transposon activity (Genetic Services Inc), and that of Arl4-GFP was targeted to the attP2 recombination site (BestGene Inc). Recovered homozygous-viable Arf4-GFP transgenic fly lines containing the transgene on the second chromosome were used for this study.

#### shRNA constructs

The shRNAs were designed based on the algorithm by Vert et al. [41]. For *arf1*, they targeted two unique sequences: shRNA2 top strand 5’- ctagcagtCTGAGGGATGCAGTCTTACTAtagttatattcaagcataTAGTAAGACTGCATCCCTCAGgcg -3'; shRNA4 top strand 5’-ctagcagtAACCTTTCAAGCAGCATATAAtagttatattcaagcataTTATATGCTGCTTGAAAGGTTgcg -3'. The constructs were ligated into the pValium22 vector (gift from the *Drosophila* Transgenic RNAi Resource Project) using the restriction enzymes Nhe1 and EcoR1, confirmed by PCR, sequenced and targeted to the attp2 recombination site on chromosome 3 for transgenic flies (BestGene Inc).

For *arl4*, they targeted two unique sequences: shRNA1 top strand 5’ctagcagtCAGGGTGTTCCCGTTCTGATAtagttatattcaagcataTATCAGAACGGGAACACCCTGgcg-3’; shRNA3 top strand 5’ctagcagtTCCCGTTCTGATACTAGCAAAtagttatattcaagcataTTTGCTAGTATCAGAACGGGAgcg-3’. The constructs were ligated into the pWalium20 vector (gift from the *Drosophila* Transgenic RNAi Resource Project) using the restriction enzymes Nhe1 and EcoR1, confirmed by PCR, sequenced and targeted to the attp2 recombination site on chromosome 2 for transgenic flies (BestGene Inc).

### Other *Drosophila* stocks and genetics

The following fly lines were used: maternal-α4-tubulin-GAL4::VP16 flies (gift of M. Peifer, University of North Carolina, Chapel Hill, USA), UASp-mCherry-shRNA flies (P[VALIUM20-mCherry]attP2, Bloomington Drosophila Stock Center BDSC #35785), UASp-Arf1-GFP [42], UASp-Arf6-GFP [43], *arf6* ^KO^ [38], *arf6* ^1^ [39], RNAi-resistant UASp-GFP-Step[WT] and UASp-GFP-Step[E173K] [14], *tubulin*-Step[PH+PB] (also known as tGPH [33]; BDSC #8164).

For UAS transgene expression, we analyzed progeny of mothers heterozygous for maternal-α4-tubulin-GAL4::VP16 and for the transgene. For arf6 mutants, we analyzed progeny of mothers *trans*-heterozygous for *arf6* ^KO^ and *arf6* ^1^.

### Probing lipids on solid supports

For the GST fusions of the wildtype and mutated Step PH domain, PCR products spanning the protein sequence in Fig 2A were cloned into the pGEX 6P vector for N-terminal GST tagging. The WT and mutated *step* sequences in the gateway entry vector were used as the templates for the PCR, using the same primers. For the GST fusion of the mouse Grp1 PH domain construct, DNA encoding the protein sequence in Fig 2A was synthesized (GenScript Inc) and cloned into the pGEX 6P vector for N-terminal GST tagging. The GST fusion proteins were purified as described previously [44]. PIP strips were probed with purified GST proteins using supplier instructions (Echelon, Salt Lake City, UT; www.echelon-inc.com/content/EBI/product/files/PROTOCOL_Strip_Array.v9.pdf). Rabbit antibodies against GST (generated in our lab), horseradish peroxidase (HRP)–conjugated secondary antibodies (Thermo Fisher Scientific, Waltham, MA), HRP detection reagents (Thermo Fisher Scientific), and a FluorChem 8900 imaging system (Alpha Innotech, Santa Clara, CA) were used to detect the GST proteins.

### Embryo Staining and Imaging

Embryos were fixed for 20 minutes in 1:1 3.7% formaldehyde in PBS:heptane and then devitellinized in methanol. Blocking and staining were in PBS containing 1% goat serum, 0.1% Triton X-100 and 1% sodium azide. Antibodies used were: rabbit, Amphiphysin (1:2000; gift of G. Boulianne, Hospital for Sick Children, Toronto, Canada); mouse, Dlg (1:100; Developmental Studies Hybridoma Bank (DSHB)), KDEL (1:500, Abcam). Secondary antibodies were conjugated to Alexa Fluor 568 and Alexa Fluor 647 (Life Technologies). Embryos were mounted in Aqua Polymount (Polysciences).

For live imaging, dechorionated embryos were glued to a cover slip using tape adhesive dissolved in heptane and mounted in halocarbon oil (series 700; Halocarbon Products). The cover slip, with the embryos facing up, was set into the bottom of a glass bottom culture dish (MatTek) with its original coverslip removed.

Immuno-fluorescent images were collected by a spinning disk confocal system (Zeiss Axiovert 200M; Quorum Technologies, Guelph, Canada) at room temperature using 10x EC Plan-Neofluar NA 0.3, 40x Plan Neofluar NA 1.3 and 63x Plan Apochromat NA 1.4 objectives (Carl Zeiss, Toronto, Canada) with a piezo top plate and an EM CCD camera (Hamamatsu C9100-13; Hamamatsu Photonics, Hamamatsu, Japan), where z-stacks had 1μm and 300 nm step sizes, respectively. These images were analyzed with Volocity software (PerkinElmer, Waltham, USA).

For fluorescence loss in photobleaching (FLIP) experiments, cells were photobleached for 5 sec with an argon laser at the furrow tip level 1 min intervals. The bleach region was a long rectangular box with a length of 48 μm and a width of 1.3 μm across the field of view, spanning about 10 furrows. Cells were imaged for 20–30 sec before photobleaching and up to 10 min after the photobleaching began.

Photoshop (Adobe, Mountain View, CA) was used for figure preparation. Except where noted, input levels were adjusted so the main signal range spanned the entire output grayscale. Images were resized by bicubic interpolation without noticeable changes at normal viewing magnifications.

### Post-acquisition image analysis and quantification

#### Quantifications of membrane and cytoplasmic GFP-Step levels

For each embryo that had furrow lengths between 2 to 7 μm, the plot profile tool in Image J was used to measured across 10–15 cellularization furrows, and the five highest and lowest values were selected across the distribution. These values were averaged to calculate an overall PM:cytoplasm ratio for each embryo.

#### FLIP analyses of GFP-Step constructs

For each embryo, the fluorescence intensity of the GFP-Step construct was measured at three distinct furrows of second row of cells away from those being photobleached. For these measurements, the plot profile tool was used to collect the average fluorescence intensity along the furrow region of interest, and to subtract cytosolic background from this value we subtracted the average fluorescence intensity of a circle with a diameter of 2μm at the centre of one of the cells. To correct for photobleaching from the imaging laser, we determined the average fluorescence intensity in a long rectangular box with a width of 5 μm and a length of 48 μm, at least 27 μm away from the repeatedly photobleached region, and divided the background corrected furrow values by these amounts at each time point.

### Statistics

Comparisons were done using Student’s t-tests. Means are shown with standard deviations.
